# Supplementary material for: Effects of Individualised High Positive End-Expiratory Pressure and Crystalloid Administration on Postoperative Pulmonary Function in Patients Undergoing Robotic-Assisted Radical Prostatectomy: A Prospective Randomised Single-Blinded Pilot Study
Source: J Clin Med. 2023 Feb 12;12(4):1460. doi: 10.3390/jcm12041460 (PMC9960679; doi:10.3390/jcm12041460)
Supplement: Supplementary file 1 [file jcm-12-01460-s001.zip › Table S1 030223.pdf]

Table S1: Mechanical ventilation, blood gas and haemodynamic parameters at five time points during RALP (n=98).

**Parameters**		**$PEEP_5$ and liberal volume (n=23)**	**$PEEP_5$ and restrictive volume (n=27)**	**$PEEP_{IND}$ and liberal volume (n=24)**	**$PEEP_{IND}$ and restrictive volume (n=24)**	**P-value**
**Minute volume** (litres min^{-1})	**T1**	7.3 (± 0.9)	6.9 (± 0.7)	6.7 (± 0.8)	6.8 (± 1.1)	0.055
	**T2**	8.2 (± 1.1)	8.1 (± 0.9)	7.7 (± 1.0)	8.0 (± 1.1)	0.323
	**T3**	8.6 (± 1.2)	8.1 (± 1.0)	8.2 (± 1.1)	8.3 (± 1.6)	0.574
	**T4**	8.7 (± 2.0)	8.2 (± 1.0)	8.1 (± 1.1)	8.1 (± 1.2)	0.370
	**T5**	8.6 (± 2.1)	8.4 (± 1.3)	8.1 (± 1.6)	8.3 (± 1.6)	0.735
**Tidal volume / PBW** (ml/kg)	**T1**	**8.3 (± 0.9)**	**7.7 (± 0.5)**	**8.0 (± 0.7)**	**7.7 (± 0.8)**	**0.010**
	**T2**	7.6 (± 0.9)	7.7 (± 0.7)	7.6 (± 1.0)	7.5 (± 1.0)	0.875
	**T3**	7.9 (± 0.9)	7.5 (± 0.7)	7.6 (± 1.1)	7.5 (± 1.0)	0.567
	**T4**	7.9 (± 1.3)	7.5 (± 0.7)	7.4 (± 1.2)	7.7 (± 1.1)	0.524
	**T5**	8.1 (± 1.3)	7.9 (± 0.7)	8.2 (± 0.7)	7.9 (± 0.8)	0.508
**PEEP** (cmH_2O)	**T1**	5 (± 0)	5 (± 0)	5 (± 0)	5 (± 0)	0.357
	**T2**	**5 (± 0)**	**5 (± 0)**	**15.2 (± 1.5)**	**15.6 (± 2.0)**	**<0.001**
	**T3**	**5 (± 0)**	**5 (± 0)**	**15.1 (± 1.6)**	**15.6 (± 2.0)**	**<0.001**
	**T4**	**5 (± 0)**	**5 (± 0)**	**15.1 (± 1.7)**	**15.6 (± 2.0)**	**<0.001**
	**T5**	**5 (± 0)**	**5 (± 0)**	**15.1 (± 1.6)**	**15.6 (± 2.0)**	**<0.001**
**Plateau pressure** (cmH_2O)	**T1**	15.0 (± 2.3)	14.6 (± 2.0)	14.3 (± 2.0)	14.1 (± 1.6)	0.735
	**T2**	**26.0 (± 4.0)**	**24.5 (± 5.3)**	**27.6 (± 3.1)**	**28.8 (± 2.8)**	**0.001**
	**T3**	**26.7 (± 4.4)**	**26.0 (± 4.7)**	**29.3 (± 3.5)**	**28.2 (± 3.0)**	**0.015**
	**T4**	**27.6 (± 4.6)**	**25.9 (± 3.5)**	**29.6 (± 3.4)**	**29.5 (± 2.5)**	**0.001**
	**T5**	**17.0 (± 1.9)**	**16.1 (± 2.1)**	**24.0 (± 2.3)**	**25.0 (± 2.6)**	**<0.001**
**Driving pressure** (cmH_2O)	**T1**	10.2 (± 2.2)	9.9 (± 2.3)	9.7 (± 2.1)	9.5 (± 1.7)	0.711
	**T2**	**21.4 (± 4.0)**	**19.9 (± 5.4)**	**13.6 (± 2.7)**	**13.5 (± 2.7)**	**<0.001**
	**T3**	**22.0 (± 4.2)**	**21.2 (± 4.8)**	**14.7 (± 3.5)**	**12.9 (± 2.8)**	**<0.001**
	**T4**	**23.0 (± 4.5)**	**21.6 (± 3.5)**	**15.0 (± 2.9)**	**14.3 (± 2.7)**	**<0.001**
	**T5**	**12.4 (± 1.8)**	**11.5 (± 2.2)**	**9.2 (± 1.4)**	**9.6 (± 1.8)**	**<0.001**
**Dynamic lung compliance** (ml cm H_2O^{-1})	**T1**	80.7 (± 18.7)	79.7 (± 21.2)	82.2 (± 21.0)	80.9 (± 18.9)	0.979
	**T2**	**31.6 (± 7.7)**	**37.0 (± 13.3)**	**48.3 (± 14.7)**	**57.4 (± 19.7)**	**<0.001**
	**T3**	**31.5 (± 7.8)**	**33.9 (± 9.9)**	**45.6 (± 13.8)**	**55.3 (± 13.4)**	**<0.001**
	**T4**	**29.7 (± 8.2)**	**30.8 (± 7.3)**	**44.4 (± 15.6)**	**51.8 (± 15.3)**	**<0.001**
	**T5**	**67.5 (± 28.4)**	**70.5 (± 20.3)**	**91.9 (± 31.8)**	**89.1 (± 14.3)**	**<0.001**

**Mechanical power #** (J/min)	**T1**	10.0 (±1.9)	9.1 (± 1.4)	9.1 (± 1.4)	9.0 (± 1.9)	0.099
	**T2**	**17.4 (± 3.4)**	**16.2 (± 3.9)**	**23.4 (± 4.0)**	**24.0 (± 3.5)**	**<0.001**
	**T3**	**18.5 (± 3.6)**	**16.9 (± 3.5)**	**25.4 (± 3.9)**	**24.5 (± 5.0)**	**<0.001**
	**T4**	**19.3 (± 5.2)**	**17.2 (± 3.3)**	**25.2 (± 3.9)**	**25.0 (± 3.8)**	**<0.001**
	**T5**	**13.1 (± 3.6)**	**12.0 (± 2.1)**	**22.1 (± 4.9)**	**23.0 (± 5.5)**	**<0.001**
**pH**	**T1**	7.40 (± 0.03)	7.40 (± 0.03)	7.41 (± 0.03)	7.41 (± 0.03)	0.299
	**T2**	7.37 (± 0.02)	7.37 (± 0.03)	7.37 (± 0.04)	7.38 (± 0.03)	0.631
	**T3**	7.37 (± 0.03)	7.37 (± 0.03)	7.36 (± 0.04)	7.37 (± 0.04)	0.874
	**T4**	7.36 (± 0.04)	7.36 (± 0.04)	7.35 (± 0.05)	7.35 (± 0.04)	0.980
	**T5**	7.35 (± 0.04)	7.34 (± 0.04)	7.34 (± 0.04)	7.34 (± 0.04)	0.848
**$PaCO_2$** (mmHg)	**T1**	39.0 (± 3.5)	37.9 (± 3.5)	37.5 (± 3.1)	37.5 (± 3.4)	0.406
	**T2**	40.7 (± 2.9)	39.6 (± 3.1)	41.0 (± 4.0)	39.9 (± 3.4)	0.406
	**T3**	41.7 (± 3.9)	40.1 (± 3.6)	41.5 (± 4.2)	40.8 (± 4.2)	0.462
	**T4**	42.5 (± 5.6)	41.2 (± 3.1)	42.3 (± 4.3)	42.4 (± 4.9)	0.713
	**T5**	42.5 (± 5.5)	42.6 (± 4.3)	41.9 (± 4.7)	42.0 (± 5.7)	0.957
**End-tidal CO_2** (mmHg)	**T1**	33.5 (± 2.0)	33.0 (± 2.8)	31.8 (± 1.7)	32.6 (± 2.1)	0.073
	**T2**	35.8 (± 2.6)	35.4 (± 2.3)	36.1 (± 3.3)	35.5 (± 2.9)	0.821
	**T3**	37.0 (± 3.8)	35.7 (± 2.7)	36.0 (± 3.0)	36.6 (± 2.8)	0.502
	**T4**	37.4 (± 4.6)	36.3 (± 3.1)	36.0 (± 3.2)	37.5 (± 3.8)	0.348
	**T5**	39.1 (± 4.0)	39.0 (± 3.1)	38.8 (± 4.2)	39.8 (± 3.5)	0.777
**PaO_2/FiO_2 ratio** (mmHg)	**T1**	420 (± 93)	402 (± 115)	428 (± 112)	395 (± 118)	0.715
	**T2**	**329 (± 100)**	**334 (± 94)**	**408 (± 87)**	**425 (± 86)**	**<0.001**
	**T3**	**316 (± 103)**	**320 (± 103)**	**398 (± 92)**	**397 (± 90)**	**0.002**
	**T4**	**315 (± 99)**	**315 (± 87)**	**382 (± 93)**	**380 (± 82)**	**0.007**
	**T5**	**339 (± 90)**	**336 (± 83)**	**395 (± 83)**	**413 (± 76)**	**0.002**
**SpO2** (%)	**T1**	99.6 (± 0.6)	99.4 (± 1.3)	99.6 (± 1.0)	98.8 (± 2.1)	0.168
	**T2**	**98.4 (± 1.3)**	**98.0 (± 1.6)**	**99.6 (± 0.7)**	**98.8 (± 1.4)**	**<0.001**
	**T3**	**97.8 (± 1.5)**	**97.6 (± 1.7)**	**99.0 (± 1.2)**	**98.7 (± 1.3)**	**<0.001**
	**T4**	**97.7 (± 1.7)**	**97.3 (± 1.5)**	**98.8 (± 1.2)**	**98.3 (± 1.3)**	**<0.001**
	**T5**	**98.7 (± 1.5)**	**98.1 (± 1.4)**	**99.5 (± 1.1)**	**98.8 (± 1.4)**	**0.006**
**Respiratory rate** (n/min)	**T1**	12.2 (± 0.7)	12.2 (± 1.0)	11.8 (± 1.1)	12.1 (± 1.2)	0.586
	**T2**	14.9 (± 1.6)	14.3 (± 1.4)	14.5 (± 2.2)	14.8 (± 2.3)	0.657
	**T3**	15.0 (± 1.6)	14.7 (± 1.5)	15.3 (± 1.8)	15.1 (± 2.1)	0.700
	**T4**	15.2 (± 1.9)	14.8 (± 1.7)	15.6 (± 2.0)	14.8 (± 2.0)	0.414
	**T5**	14.6 (± 2.6)	14.4 (± 1.7)	14.0 (± 2.7)	14.6 (± 2.1)	0.782

**Mean arterial pressure** (mmHg)	**T1**	74.5 (± 11.0)	78.6 (± 12.4)	77.4 (± 14.5)	79.1 (± 20.7)	0.726
	**T2**	103.4 (± 10.7)	101.2 (± 10.8)	104.3 (± 15.1)	101.1 (± 10.8)	0.728
	**T3**	99.0 (± 11.3)	96.6 (± 11.9)	97.4 (± 11.9)	96.8 (± 13.7)	0.900
	**T4**	**96.3 (± 11.0)**	**89.1 (± 10.0)**	**94.0 (± 11.8)**	**87.7 (± 11.8)**	**0.028**
	**T5**	82.5 (± 10.8)	78.9 (± 10.6)	74.6 (± 8.1)	78.8 (± 11.2)	0.077
**Noradrenaline** (mg/h)	**T1**	0.13 (± 0.11)	0.14 (± 0.08)	0.15 (± 0.09)	0.13 (± 0.15)	0.897
	**T2**	0 (± 0)	0.01 (± 0.04)	0.01 (± 0.03)	0.01 (± 0.04)	0.601
	**T3**	0 (± 0)	0 (± 0.02)	0.01 (± 0.02)	0.02 (± 0.04)	0.105
	**T4**	0 (± 0)	0.01 (± 0.03)	0 (± 0.02)	0.01 (± 0.05)	0.327
	**T5**	**0.16 (± 0.11)**	**0.21 (± 0.13)**	**0.23 (± 0.13)**	**0.29 (± 0.17)**	**0.010**

Notes: $p<0.05$: in bold; entries depict the mean (SD), and p-values compare the four arms using repeated-measures ANOVA; $PaCO_2$: partial pressure of carbon dioxide in arterial blood; PBW: predicted body weight; SpO_2: peripheral oxygen saturation;T1: 5 min after intubation in supine position, T2: 30 min after the start of pneumoperitoneum and Trendelenburg position, T3: 60 min after the start of pneumoperitoneum and Trendelenburg position, T4: 90 min after the start of pneumoperitoneum and Trendelenburg position, T5: before extubation in supine position
