# Supplementary material for: Effects of Individualised High Positive End-Expiratory Pressure and Crystalloid Administration on Postoperative Pulmonary Function in Patients Undergoing Robotic-Assisted Radical Prostatectomy: A Prospective Randomised Single-Blinded Pilot Study
Source: J Clin Med. 2023 Feb 12;12(4):1460. doi: 10.3390/jcm12041460 (PMC9960679; doi:10.3390/jcm12041460)
Supplement: Supplementary file 1 [file jcm-12-01460-s001.zip › Table S2 010223.pdf]

Table S2: Spirometric parameters compared between PEEP groups at four time points: on the day before surgery, in the recovery room and on the first and second postoperative day (n=98)

Parameters	Time point	$PEEP_5$ (n=50)	$PEEP_{IND}$ (n=48)	P-value
**VC** (litre)	Preoperative day	4.46 (± 0.9)	4.39 (± 0.7)	0.610
	Recovery room	3.32 (± 0.9)	3.31 (± 0.8)	0.993
	Postoperative day 1	3.48 (± 0.8)	3.52 (± 0.7)	0.843
	Postoperative day 2	3.83 (± 0.7)	3.78 (± 0.7)	0.726
**FVC** (litre)	Preoperative day	4.32 (± 0.8)	4.16 (± 0.6)	0.294
	Recovery room	3.45 (± 0.9)	3.45 (± 0.8)	0.986
	Postoperative day 1	3.46 (± 0.8)	3.50 (± 0.7)	0.803
	Postoperative day 2	3.79 (± 0.8)	3.70 (± 0.7)	0.532
**FEV1** (litre)	Preoperative day	3.16 (± 0.6)	3.13 (± 0.5)	0.811
	Recovery room	2.42 (± 0.6)	2.49 (± 0.6)	0.600
	Postoperative day 1	2.54 (± 0.6)	2.71 (± 0.6)	0.151
	Postoperative day 2	2.74 (± 0.6)	2.83 (± 0.6)	0.457
**FEV_1/FVC ratio (Tiffeneau index)** (%)	Preoperative day	73.4 (± 7.6)	75.8 (± 8.1)	0.224
	Recovery room	70.7 (± 10.2)	72.0 (± 8.5)	0.484
	Postoperative day 1	**73.6 (± 8.8)**	**77.6 (± 6.6)**	**0.014**
	Postoperative day 2	**72.7 (± 9.3)**	**76.5 (± 6.1)**	**0.021**
**PEF** (litre/min)	Preoperative day	471.1 (± 114.3)	455.2 (± 120.1)	0.502
	Recovery room	299.2 (± 104.9)	292.6 (± 98.6)	0.749
	Postoperative day 1	361.6 (± 115.4)	381.0 (± 99.9)	0.377
	Postoperative day 2	372.0 (± 113.2)	388.4 (± 106.8)	0.466

**FEF_{25}** (litre/sec)	Preoperative day	6.35 (± 1.8)	6.42 (± 1.9)	0.849
	Recovery room	4.51 (± 1.8)	4.34 (± 1.6)	0.617
	Postoperative day 1	4.99 (± 1.9)	5.68 (± 1.6)	0.057
	Postoperative day 2	5.27 (± 1.9)	5.72 (± 1.7)	0.220
**FEF_{50}** (litre/sec)	Preoperative day	3.39 (± 1.3)	3.77 (± 1.4)	0.172
	Recovery room	2.57 (± 1.2)	2.67 (± 1.1)	0.664
	**Postoperative day 1**	**2.73 (± 1.1)**	**3.36 (± 1.1)**	**0.005**
	**Postoperative day 2**	**2.88 (± 1.1)**	**3.49 (± 1.4)**	**0.018**
**FEF_{75}** (litre/sec)	Preoperative day	0.89 (± 0.5)	1.05 (± 0.5)	0.098
	Recovery room	0.72 (± 0.4)	0.85 (± 0.4)	0.119
	Postoperative day 1	0.78 (± 0.4)	0.96 (± 0.5)	0.061
	Postoperative day 2	0.81 (± 0.5)	0.92 (± 0.4)	0.227
**FEF_{25-75}** (litre/sec)	Preoperative day	2.36 (±1.0)	2.65 (± 0.9)	0.139
	Recovery room	1.80 (± 0.8)	1.98 (± 0.8)	0.273
	**Postoperative day 1**	**1.95 (± 0.8)**	**2.41 (± 0.9)**	**0.009**
	**Postoperative day 2**	**2.07 (± 0.8)**	**2.45 (± 0.9)**	**0.033**

Notes: $P<0.05$: in bold; Entries depict the mean (SD), and P-values compare the PEEP groups using a repeated-measures t-test; FEF_{25-75}: mean forced expiratory flow; FEF_{25}: forced expiratory flow after one quarter of FVC; FEF_{50}: forced expiratory flow after half of FVC; FEF_{75}: forced expiratory flow after three quarters of FVC; FEV_1: forced expiratory volume in 1 second; FVC: forced vital capacity; PEEP: positive end-expiratory pressure; PEF: peak expiratory flow; VC: vital capacity
