# Supplementary material for: Effects of Individualised High Positive End-Expiratory Pressure and Crystalloid Administration on Postoperative Pulmonary Function in Patients Undergoing Robotic-Assisted Radical Prostatectomy: A Prospective Randomised Single-Blinded Pilot Study
Source: J Clin Med. 2023 Feb 12;12(4):1460. doi: 10.3390/jcm12041460 (PMC9960679; doi:10.3390/jcm12041460)
Supplement: Supplementary file 1 [file jcm-12-01460-s001.zip › Table S3 010223.pdf]

Table S3: Spirometric parameters compared between crystalloid groups at four time points: on the day before surgery, in the recovery room and on the first and second postoperative day (n=98)

Parameters	Time point	Liberal crystalloids (n=47)	Restrictive crystalloids (n=51)	P-value
**VC** (litre)	Preoperative day	4.60 (± 0.7)	4.62 (± 0.8)	0.887
	Recovery room	3.34 (± 0.8)	3.29 (± 0.9)	0.803
	Postoperative day 1	3.45 (± 0.8)	3.54 (± 0.7)	0.588
	Postoperative day 2	3.82 (± 0.7)	3.80 (± 0.7)	0.911
**FVC** (litre)	Preoperative day	4.41 (± 0.7)	4.41 (± 0.8)	0.984
	Recovery room	3.47 (± 0.8)	3.44 (± 0.8)	0.889
	Postoperative day 1	3.43 (± 0.8)	3.53 (± 0.8)	0.538
	Postoperative day 2	3.71 (± 0.7)	3.78 (± 0.7)	0.656
**FEV1** (litre)	Preoperative day	3.34 (± 0.6)	3.33 (± 0.6)	0.918
	Recovery room	2.48 (± 0.6)	2.43 (± 0.6)	0.676
	Postoperative day 1	2.61 (± 0.6)	2.63 (± 0.6)	0.832
	Postoperative day 2	2.79 (± 0.6)	2.78 (± 0.6)	0.971
**FEV_1/FVC ratio (Tiffeneau index)** (%)	Preoperative day	75.9 (± 7.2)	75.7 (± 6.3)	0.905
	Recovery room	72.1 (± 9.5)	70.6 (± 9.3)	0.422
	Postoperative day 1	76.3 (± 7.7)	74.8 (± 8.4)	0.348
	Postoperative day 2	75.3 (± 6.9)	74.0 (± 9.0)	0.430
**PEF** (litre/min)	Preoperative day	502.7 (± 122.0)	506.5 (± 114.5)	0.872
	Recovery room	313.5 (± 108.1)	279.8 (± 93.1)	0.100
	Postoperative day 1	383.1 (± 111.1)	360.2 (± 104.8)	0.296
	Postoperative day 2	382.8 (± 116.5)	377.6 (± 104.5)	0.817

**FEF_{25}** (litre/sec)	Preoperative day	6.76 (± 1.9)	6.89 (± 1.7)	0.709
	Recovery room	4.64 (± 1.7)	4.23 (± 1.7)	0.227
	Postoperative day 1	5.46 (± 1.8)	5.21 (± 1.8)	0.491
	Postoperative day 2	5.50 (± 1.7)	5.50 (± 1.9)	0.967
**FEF_{50}** (litre/sec)	Preoperative day	3.84 (± 1.4)	3.83 (± 1.5)	0.978
	Recovery room	2.72 (± 1.1)	2.52 (± 1.1)	0.401
	Postoperative day 1	3.11 (± 1.1)	2.97 (± 1.1)	0.558
	Postoperative day 2	3.20 (± 1.2)	3.20 (± 1.3)	0.873
**FEF_{75}** (litre/sec)	Preoperative day	1.22 (± 1.0)	1.11 (± 0.7)	0.547
	Recovery room	0.76 (± 0.4)	0.80 (± 0.4)	0.600
	Postoperative day 1	0.86 (± 0.4)	0.88 (± 0.5)	0.786
	Postoperative day 2	0.88 (± 0.4)	0.85 (± 0.5)	0.781
**FEF_{25-75}** (litre/sec)	Preoperative day	2.79 (± 1.0)	2.71 (± 1.1)	0.721
	Recovery room	1.91 (± 0.8)	1.86 (± 0.8)	0.731
	Postoperative day 1	2.20 (± 0.9)	2.15 (± 0.9)	0.776
	Postoperative day 2	2.28 (± 0.8)	2.24 (± 0.9)	0.828

Notes: $P<0.05$: in bold; Entries depict the mean (SD), and P-values compare the volume groups using a repeated-measures t-test; FEF_{25-75}: mean forced expiratory flow; FEF_{25}: forced expiratory flow after one quarter of FVC; FEF_{50}: forced expiratory flow after half of FVC; FEF_{75}: forced expiratory flow after three quarters of FVC; FEV_1: forced expiratory volume in 1 second; FVC: forced vital capacity; PEF: peak expiratory flow; VC: vital capacity
