# Supplementary material for: Effects of Individualised High Positive End-Expiratory Pressure and Crystalloid Administration on Postoperative Pulmonary Function in Patients Undergoing Robotic-Assisted Radical Prostatectomy: A Prospective Randomised Single-Blinded Pilot Study
Source: J Clin Med. 2023 Feb 12;12(4):1460. doi: 10.3390/jcm12041460 (PMC9960679; doi:10.3390/jcm12041460)
Supplement: Supplementary file 1 [file jcm-12-01460-s001.zip › Table S4 010223.pdf]

Table S2: Body weight and brain natriuretic peptide levels at three time points: before surgery and on the first and second postoperative day (n=98)

Parameters	Time point	$PEEP_5$ and liberal volume (n=23)	$PEEP_5$ and restrictive volume (n=27)	$PEEP_{IND}$ and liberal volume (n=24)	$PEEP_{IND}$ and restrictive volume (n=24)	P-value
**Body weight** (kg)	Preoperative day	89.2 (± 15.0)	84.5 (± 9.87)	82.2 (± 9.5)	82.3 (± 11.3)	0.143
	Postoperative day 1	91.0 (± 15.8)	85.8 (± 9.9)	83.8 (± 9.3)	83.7 (± 11.2)	0.134
	Postoperative day 2	89.2 (± 15.4)	84.8 (± 9.8)	82.8 (± 9.3)	82.8 (±11.3)	0.203
**BNP** (pg/ml)	Preoperative day	41.6 (± 47.4)	49.7 (± 55.0)	55.4 (± 78.9)	39.9 (± 33.7)	0.755
	Postoperative day 1	73.5 (± 83.8)	64.8 (± 40.5)	94.4 (± 93.1)	76.1 (± 46.5)	0.492
	Postoperative day 2	33.3 (± 29.8)	38.5 (± 31.8)	53.4 (± 100.6)	38.7 (± 34.9)	0.662

Notes: P<0.05: in bold; Entries depict the mean (SD), and p-values compare the four arms using repeated-measures ANOVA; BNP: brain natriuretic peptide
